# Zearalenone (ZEN) and Its Metabolite Levels in Tissues of Wild Boar (*Sus scrofa*) from Southern Italy: A Pilot Study

**DOI:** 10.3390/toxins15010056

**Published:** 2023-01-09

**Authors:** Consiglia Longobardi, Sara Damiano, Gianmarco Ferrara, Serena Montagnaro, Valentina Meucci, Luigi Intorre, Samanta Bacci, Luigi Esposito, Nadia Piscopo, Antonio Rubino, Antonio Raffaele, Salvatore Florio, Roberto Ciarcia

**Affiliations:** 1 Department of Mental, Physical Health and Preventive Medicine, University of Campania “Luigi Vanvitelli”, Largo Madonna delle Grazie, 80138 Naples, Italy; 2 Department of Veterinary Medicine and Animal Productions, University of Naples “Federico II”, 80137 Naples, Italy; 3 Department of Veterinary Sciences, University of Pisa, 56124 Pisa, Italy; 4 President of the Local Management Hunting Authority (ATC), Province of Avellino, Campania Region, 83100 Avellino, Italy

**Keywords:** Zearalenone, α-Zearalenol, β-Zearalenol, wild boar, HPLC-FLD

## Abstract

Zearalenone (ZEN) is a non-steroidal estrogenic mycotoxin produced by the fungi of the *Fusarium* genera, and is a contaminant of cereals and plant products. ZEN and its metabolites are considered endocrine disruptors, and could have various toxic effects on animals and humans. In recent years, there has been a significant demographic increase in wild boar (*Sus scrofa*) in many mountainous and hilly areas of Italy, including the Campania region, mainly due to global climate change. The wild boar can be defined as a generalist and omnivorous species capable of varying its diet; therefore, it can play a role as an environmental bioindicator towards contaminants such as mycotoxins. This study was conducted to evaluate, for the first time, the concentrations of ZEN and its metabolites in the liver, kidney, and muscle of 82 wild boars shot in their habitat by hunters with hunting permits in different localities of Avellino province (Campania region, Southern Italy) from 2021 to 2022. The samples were collected and analyzed with an SPE clean-up and high-pressure liquid chromatography method with fluorescence detection. The results indicated that ZEN and α-Zearalenol were present in most of the samples, suggesting that a plan to monitor these mycoestrogens is essential to achieve the goals of “One Health”.

## 1. Introduction

The wild boar (*Sus scrofa*) is an opportunistic omnivore whose diet is highly variable and strongly influenced by environmental changes. In Europe, the crops consumed by wild boar mainly include energy-rich plant foods such as acorns, chestnuts, pine seeds, and olives, in addition to other agricultural crops, such as maize, wheat, barley, rye, oats, rice, sorghum, potatoes, and sugar beet [1]. In recent years, we have witnessed an increase in the population and habitat of wild boars in many mountainous and hilly areas of Italy, including the Campania region as a result of global climate change. Global climate change has led to an increase in temperature and humidity, which favors the development of fungi on various food crops, especially cereals [2]. Zearalenone (ZEN) is a toxic secondary metabolite produced by several *Fusarium* species that grow on crops [3]. ZEN is a mycoestrogen classified as an endocrine disruptor and, since its chemical structure is similar to the endogenous estrogen 17-estradiol, it can bind estrogen receptors [4]. This mycotoxin contaminates cereals such as wheat, barley, corn, sorghum, rye [5,6], rice [5], corn silage [7], sesame seeds, hay [8], flour, malt, soybeans, beer [9], corn oil [10], dried fruits, spices, and milk [11], and its thermostability allows it to withstand storage, milling, processing, and cooking [12]. ZEN is also known to be immunotoxic, hepatotoxic [13], nephrotoxic, hematotoxic [14], and genotoxic [15]. The International Agency for Research on Cancer (IARC) has classified ZEN as a Group 3 substance (not carcinogenic to humans) [16]. Its toxicity depends on the immune status of the organism and the state of the reproductive system (juvenile or pregnant) [17]. The liver is the major distribution organ of ZEN [18] and it is metabolized mainly by hepatic hydroxysteroid dehydrogenase into α-Zearalenol (α-ZEL) and β-Zearalenol (β-ZEL) [19]. ZEN and its metabolites exhibit estrogenic [20] and anabolic effects in several animal species, resulting in infertility, hormonal dysfunction, and hyperplasia of the reproductive tract [21,22]. Animal species that are particularly sensitive to the effects of ZEN exposure include pigs and ruminants [12]. In humans, ZEN causes premature puberty [23]. In pregnant women, long-term exposure to ZEN can result in decreased embryo survival and fetal weight, as well as decreased milk production. In men, ZEN reduces sperm count and viability, impairing spermatogenesis [24]. The European Food Safety Authority (EFSA) Panel on Contaminants in the Food Chain has established a tolerable daily intake (TDI) of 0.25 µg/kg body weight of ZEN for human consumption and published a no-observed-effect level (NOEL) of 10 µg/kg body weight of ZEN per day for pigs [25]. Since most scientific information about ZEN and its metabolites in wild boars is sourced from Poland [26,27,28], we considered it necessary to investigate the concentrations of this mycotoxin and the levels of its metabolites in the muscle, liver, and kidney of wild boar in the Campania region for the first time. The animals were hunted in different locations of the province of Avellino in the Campania region, Southern Italy, where wild boar meat is traditionally used to make typical products such as “coppa” and “salami”. It follows that monitoring the mycotoxin content of wild boar meat could be important for the protection of consumer health, since contaminated products can lead to enormous economic losses and pose risks to humans and animals.

## 2. Results

**Figure 1:**
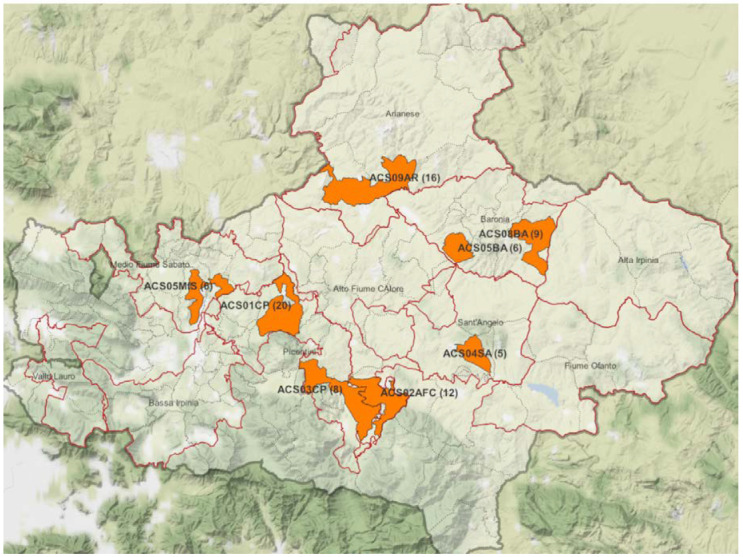
Cartographic image of the province of Avellino (Campania region, Southern Italy) with hunting areas (in orange) involved in wild boar (*Sus scrofa*) sampling.

During the study period, a total of 82 hunted wild boar samples from eight hunting areas in the province of Avellino were examined (Figure 1).

For each animal, information on gender, age, body weight, and hunting areas was recorded. For the risk factor analysis, the animals in which ZEN or one of its metabolites were detected in at least one organ were considered positive, as shown in Table 1. The samples were divided as follows: 46.3% males and 53.7% females. Young animals (0–12 months) were the most represented category (53.7%; *n* = 44), followed by older animals (>36 months) (24.4%; *n* = 20) and finally adult animals (13–36 months) (21.9%; *n* = 18). A total of 40 out of 82 wild boar samples tested (48.7%; 95% confidence interval (CI) 37.9–59.6) were positive for ZEN and/or α-ZEL. No β-ZEL was detected by HPLC in the liver, muscle, or kidney of the studied animals (Table 1). No statistical significance was demonstrated between the presence of this mycotoxin and gender, age, or body weight, although males (50.0%, 95% CI 34.1–65.9), adults (13–36 months) (61.1%, 95% CI 38.6–83.6), and those belonging to the 70–89 kg body weight class (64.3%, 95% CI 39.2–89.3) had higher percentages of ZEN and/or α-ZEL.

**Figure 2:**
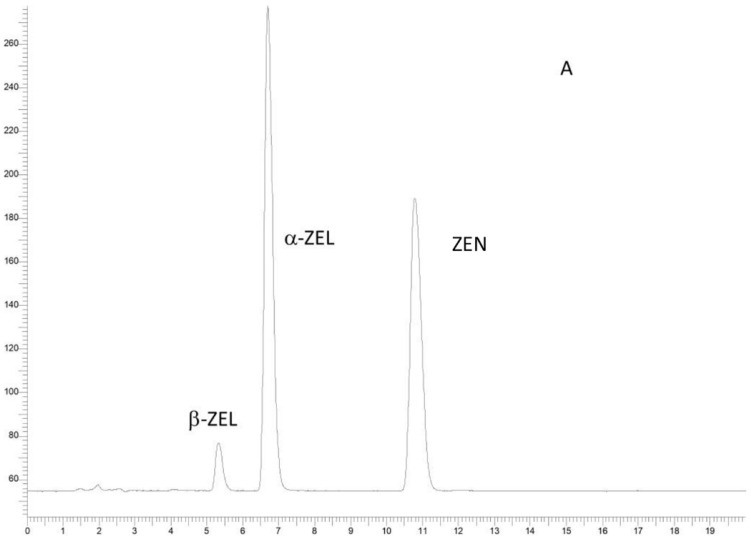

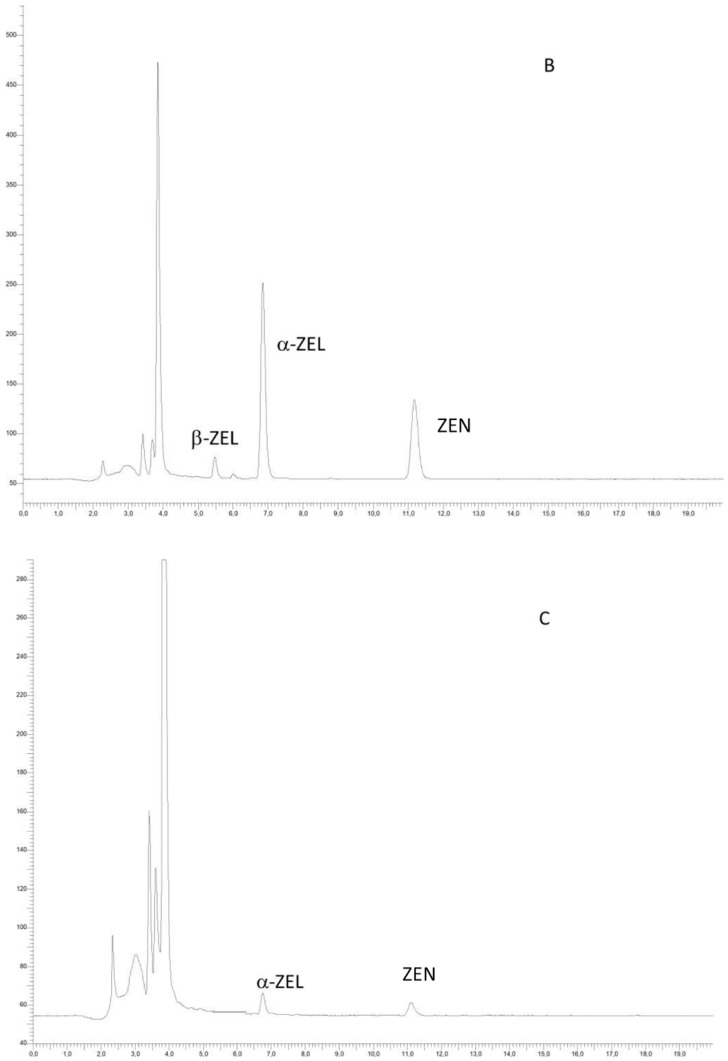

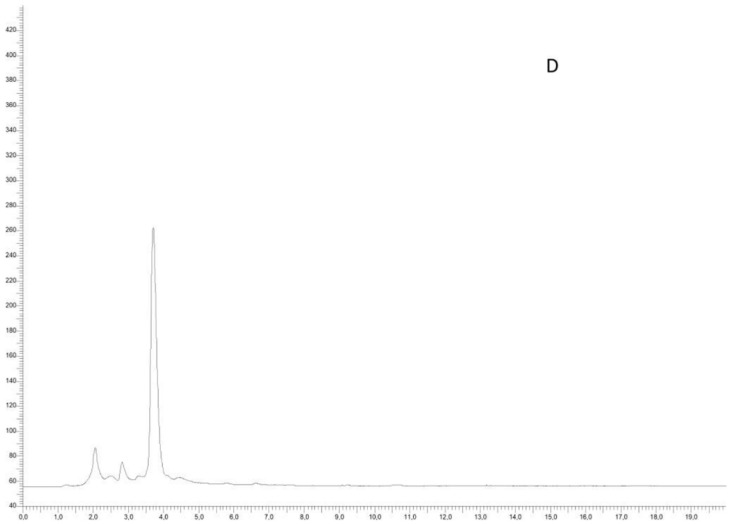
HPLC-FLD chromatograms of: (**A**) ZEN, α-ZEL and β-ZEL standard solutions (at 100 ng/mL); (**B**) a liver sample spiked with ZEN, α-ZEL and β-ZEL standard solutions (at 100 ng/mL); (**C**) a liver sample naturally contaminated with ZEN and α-ZEL; (**D**) a blank liver sample.

The results of the validation study are reported in Table S1 of the Supplementary Materials. The average recoveries were between 63% and 75% with satisfactory RSD, thus completely fulfilling the performance criteria fixed by the European Commission Regulation (2006) [29], i.e., recovery in the range of 50–120% and 70–110% for levels < 1 and between 1 and 10 ng/g, respectively. Figure 2 shows chromatograms of the liver sample of one wild boar naturally contaminated with ZEA and α-ZEL.

**Figure 3:**
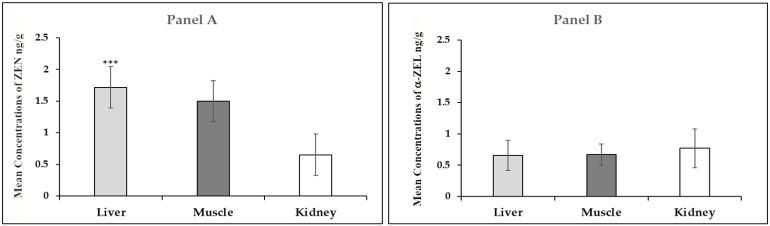
Mean of ZEN (**Panel A**) and α-ZEL (**Panel B**) concentrations (ng/g) in liver, muscle, and kidney of wild boars from Avellino Province (Campania region of Southern Italy, 2021–2022); *p*: a value of *p* < 0.05 was considered statistically significant; *** *p* < 0.001.

The mean concentrations of ZEN in the liver, muscle, and kidney samples were 1.71 ng/g, 1.49 ng/g, and 0.65 ng/g, respectively; data analysis revealed statistical significance between ZEN concentrations in the liver samples (*p* < 0.0003) (Table 2; Figure 3, Panel A). The mean α-ZEL values in the liver, muscle, and kidney samples were 0.65 ng/g, 0.66 ng/g, and 0.77 ng/g, respectively (Table 2; Figure 3, panel B). The Kruskal–Wallis test showed no statistical significance between the α-ZEL values in the liver, muscle, and kidney, although the mean α-ZEL values were higher in the kidney samples (Table 2; Figure 3, Panel B).

A weak correlation was observed between the body weight of the wild boars and the concentrations of ZEN and α-ZEL in the liver, muscle, and kidney. Similar results were found between the age of the wild boars and the concentrations of ZEN and α-ZEL in the same organs. The results are presented in Table 3.

## 3. Discussion

The problem of residue of substances with potentially toxic effects, such as mycotoxins, in foods has taken on considerable importance in terms of food safety [30]. Clearly, knowledge of the epidemiological behavior of this toxic agent is one of the key elements for planning a monitoring or management plan for this mycotoxin, which is important for public health, domestic animal health, livestock production, and wildlife conservation. To plan a management strategy, bioindicators must be identified, i.e., species that can be used to monitor exposure to toxic substances. Wild boar is an excellent species for use as a biological indicator for the detection of mycotoxins in wild meat, both because of its eating habits and because of its wide distribution. Moreover, although it is a wild species, it can be easily sampled. In the Campania region, wild boar meat is traditionally used to produce niche products, especially “coppa” and “salami”, and its liver is also highly appreciated and frequently used in local cooking recipes. Therefore, our data suggest that health surveillance of this species is needed to protect these niche products and reduce the introduction of ZEN into the human diet. As indicated in the European Community guidelines (Commission Regulation (EC) No. 401/2006) [29], control of the quality of meat intended for processing is a priority to reduce the possibility of toxin transmission to humans. In this study, ZEN and α-ZEL were detected in 48.7% (40/82; 95% CI 37.9–59.6) of the wild boars tested. No statistical significance was demonstrated between the presence of ZEN and gender, age, body weight or hunting areas, although males (50.0%, 95% CI 34.1–65.9), adults (13–36 months) (61.1%, 95% CI 38.6–83.6), and animals in the 70–89 kg body weight class (64.3%, 95% CI 39.2–89.3) had higher percentages of ZEN and α-ZEL. Thus, it seems that young animals are more sensitive than adult and heavier animals. This result may be due to the fact that wild boars with a higher body mass have greater degradation capacity [31] and access to less contaminated food sources in relation to social behavior and feeding hierarchy [32]. The primary route by which ZEN enters organisms is through the consumption of contaminated food. Our results show that ZEN and α-ZEL are mainly found in liver and muscle, and less frequently found in the kidneys. The highest concentration of ZEN was found in liver (mean = 1.71 ng/g) and this value was statistically significant (*p* = 0.0003) compared with the concentrations of ZEN in muscles (mean = 1.49 ng/g) and kidneys (mean = 0.65 ng/g). This may be related to the fact that liver and muscle are the most widely distributed organs and also to the fact that they are produced by liver microsomes, as is the case in pigs [7]. The influence of ZEN on wild boars is poorly studied. In the published data, considerably more information can be found on ZEN mycotoxicosis in domestic pigs, which are highly sensitive to ZEN [33,34]. No statistical significance was demonstrated for the concentration of the metabolite α-ZEL in the studied samples (liver, muscle, and kidney), although the highest concentrations were found in the kidney (mean = 0.770 ng/g). A possible explanation for the higher α-ZEL concentrations in the kidney than ZEN could be related to the metabolic processes of the organism. In this respect, further molecular and biochemical studies are necessary to clarify the mechanisms of ZEN toxicity in wild boar. Finally, the data found in this study showed no correlation between the concentrations of ZEN and α-ZEL in different tissues in relation to the body weight and age of the studied wild boar. Although the concentrations of ZEN and α-ZEL were not statistically significant, wild boar has proven to be an excellent biological indicator for the presence of mycotoxin in food, which leads us to infer that this mycotoxin is widely distributed in the environment. This is a global problem that leads to livestock disease, serious economic loss, and a negative impact on human health.

## 4. Conclusions

This work supports the concept that animal-derived products, particularly wild boar meat, are a potential source of ZEN and its metabolites, and that wild boars serve as sentinels for the presence of mycotoxins in the environment and crops. We detected ZEN and metabolites in almost 50% of the tested animals, with no variations based on age, gender, or region, demonstrating the extensive contamination of the collected animals. Our research improves our knowledge of mycotoxin contamination in wild boar meat and offal, demonstrating how mycotoxins, such as ZEN, can enter the human food chain in a variety of ways, posing a significant public health risk. To achieve this goal, a health monitoring plan that includes identifying mycotoxins in wild boars should be implemented.

## 5. Materials and Methods

### 5.1. Ethical Approval

No ethics committee approval was required for because the wild boars were harvested by hunters, and thus not culled for research purposes. These animals were legally killed in their own habitat by licensed hunters under the 2021–2022 annual hunting plan, approved by the Province of Avellino in the Campania region of Southern Italy.

### 5.2. Sampling Area

The Campania region is in the southern Italian peninsula, with a total area of 13,595 km^2^ and a coastline of 350 km (217 mi) bordering the Tyrrhenian Sea. The region has a temperate Mediterranean climate with cold winters and dry summers [35]. Wild boars occur in 40% of the regional territory (except for Cilento National Park, Vallo di Diano, and Alburni), and Avellino is the province with the highest percentage, followed by Salerno, Caserta, and Benevento (Figure 3). The study was carried out in eight hunting areas distributed throughout the province of Avellino (Table 1).

### 5.3. Reagents

ZEN, α-ZEL, and β-ZEL reference standards were purchased from Merck (Milan, Italy). Working solutions were prepared by diluting the stock solution with a mobile phase consisting of water/acetonitrile/methanol 50/46/4% *v*/*v*. HPLC-grade water, methanol (CH_3_OH), and acetonitrile (CH_3_CN) were purchased from VWR (Milan, Italy).

### 5.4. Chromatographic Method

The chromatographic system consisted of a PerkinElmer 200 series variable flow pump (PerkinElmer, Waltham, MA, USA) connected to a Jasco 1521 fluorescence detector (Jasco, Tokyo, Japan). The excitation wavelength (λex) and emission wavelength (λem) were set to 274 and 440 nm, respectively. The system was controlled via a PerkinElmer interface module (NCI 900 Network Chromatography Interface), and chromatograms were processed using PerkinElmer TotalChrom Navigator software. An X-Bridge C18 5 µm 250 × 4.6 mm chromatography column (Waters, Milford, MA, USA) was used. Analyses were performed at room temperature with a flow rate of 1 mL/min and an injection volume of 100 μL was used. The mobile phase consisted of water/acetonitrile/methanol 50/46/4% *v*/*v*.

### 5.5. Sample Preparation

Samples of muscle, liver, and kidney (2 g) were collected from each wild boar and homogenized for several minutes with 10 M CH_3_CN in an Ultra Turrax T25 homogenizer. The homogenizate was shaken for 10 min, the extracts were centrifuged at 4000 rpm at 4 °C for 10 min, and the supernatant was collected. The supernatant was concentrated to 2 mL by evaporation at 50 °C under a stream of nitrogen. The concentrate was mixed with 8 mL of water and the solution was applied to an Oasis HLB cartridge (60 mg, 3 cm^3^, Waters, USA) at a flow rate of 0.5 mL/min. The cartridge was previously conditioned with 2 mL CH_3_OH and 2 mL water. After washing with 2 mL of water, mycotoxins were eluted with 4 mL of CH_3_OH, and the eluate was evaporated to dryness at 50 °C. The residue was redissolved in 500 μL of HPLC mobile phase, and 100 μL of the final extract was injected into the HPLC system. Samples spiked before extraction were used to verify the performance of the extraction and purification procedure and to obtain validation parameters. Spiking solutions of ZEN and metabolites were prepared daily by dilution with the HPLC mobile phase. For the muscle, kidney, and liver samples, the spiked homogenate was left at room temperature for at least 2 h after thorough mixing for 30 min to allow equilibration, and was used to check the purification procedure before HPLC analysis.

### 5.6. Method Validation

The HPLC-FLD method was validated according to the European Commission (2002) by evaluating specificity, recovery, linearity, LOD and LOQ, repeatability and reproducibility. Several wild boar meat samples were analyzed to verify the absence of the target analyte and potential interfering compounds; then, 30 blank samples were pooled and used for the validation study. The linearity was evaluated by spiking muscle, liver, and kidney samples with ZEN, α-ZEL, and β-ZEL at 0.25, 0.5, 1, 2.5, and 5 ng/g and analyzing them using the extraction and HPLC-FLD method. The experiment was repeated three times. The repeatability was tested by analyzing the muscle, liver, and kidney samples spiked with ZEN, α-ZEL, and β-ZEL at the levels of 0.1 ng/g, 1 ng/g, and 5 ng/g. All samples were measured in triplicate on the same day. For the within-laboratory reproducibility test, each of the contamination levels was tested in triplicate over a period of five days. Repeatability and reproducibility were given as the mean of the concentrations for three and six, respectively, fortification levels were determined at three different times, and the relative standard deviation was computed as %RSD = (standard deviation/mean concentration) × 100. The results of these experiments were also used for the determination of the recovery. The LOD and LOQ were determined by the signal-to-noise approach, defined at levels resulting in signal-to-noise ratios of 3 and 10, respectively. The analytical response and chromatographic noise were measured from the chromatogram of a blank sample extract (1 mL) to which ZEN, α-ZEL, and β-ZEL solutions were added.

### 5.7. Statistical Analysis

Statistical analysis was performed using GraphPad Prism version 7 software (GraphPad Software Inc, La Jolla, CA, USA). All data were tested for normality using the Kolmogorov–Smirnov test. Since the data were not normally distributed, the Kruskal–Wallis test was used to evaluate the significance of differences between mycotoxin concentrations in different tissues. Spearman correlation coefficient analysis was used to evaluate the correlation between the concentrations of ZEN and α-ZEL in muscle, liver, and kidney, as well as the body weight of the wild boar (Med Calc). The ranges of correlation strength were r ≥ 0.8, 0.6 ≤ r < 0.8, 0.3 ≤ r ≤ 0.5, and r ≤ 0.2 for strong, moderately strong, moderate, and weak correlation, respectively. A value of *p* < 0.05 was considered statistically significant. MedCalc Statistical Software version 20.118 (MedCalc Software (free trial), Ostend, Belgium; www.medcalc.org; 2022) was used to compare proportions of positivity in relation to categorical dependent variables and to determine statistical significance within each class (gender, body weight, age, and location). Chi-square tests were used to compare the proportions of positivity related to categorically dependent variables and to determine the statistical significance within each class (gender, body weight, age, and location). Variables associated with the presence of ZEN and its metabolites were entered into binary logistic models using JMP Pro version 15.0.0 (SAS Institute Inc., Campus Drive, Cary, NC, USA). For the risk factor analysis, wild boar in which ZEN or one of its metabolites were detected in at least one organ sample were considered positive. *p* < 0.05 was considered significant. Significant differences between categories were quantified by calculating odds ratios (OR) and 95% confidence intervals (CIs).

## Figures and Tables

**Table 1:** ZEN and α-ZEL prevalence and risk factor analysis in wild boars from Avellino Province (Campania region of Southern Italy, 2021–2022).

Wild Boars	*n*	Positive	%	SE%	95% CI	X^2^	*p*	OR	95% CI
Total	82	40	48.7	10.8	37.9–59.6	-	-	-	-
Gender									
Female	44	21	47.7	14.7	32.9–62.4				
						0.04	0.9871	0.9130	0.38–2.17
Male	38	19	50.0	15.9	34.1–65.9				
Age									
0–12 months	44	21	47.7	14.7	32.9–62.5			Ref.	
13–36 months	18	11	61.1	22.5	38.6–83.6	1.732	0.4206	0.581	0.19–1.77
>36 months	20	8	40.0	21.5	18.5–61.5			1.36	0.46–4.00
Body Weight									
30–49 kg	34	17	50.0	16.8	33.2–66.8			Ref	
50–69 kg	14	6	42.8	25.9	16.9–68.7	2.181	0.5357	0.75	0.21–2.62
70–89 kg	14	9	64.3	25.1	39.2–89.3			1.8	0.49–6.49
≥90 kg	20	8	40.0	21.5	18.5–61.5			0.66	0.21–2.04
Hunting areas									
ACS01CP	20	9	45.0	21.8	23.2–66.8			Ref.	
ACS03CP	8	3	37.5	33.5	3.95–71.1			0.73	0.13–3.93
ACS02AFC	12	5	41.6	27.8	13.7–69.5	3.103	0.875	0.87	0.20–3.70
ACS04SA	5	3	60.0	42.9	17.1–100.0			1.83	0.24–13.46
ACS05MFS	6	2	33.3	37.7	0.0–71.1			0.61	0.09–4.13
ACS09AR	16	9	56.2	24.3	31.9–80.6			1.57	0.41–5.9
ACS05BA	6	3	50.0	40.0	9.9–90.0			1.22	0.19–7.59
ACS08BA	9	6	66.7	30.8	35.8–97.4			2.44	0.47–12.6

SE: standard error; 95% CI: 95% confidence interval; OR: odds ratio; Ref: reference category; *p*: *p* < 0.05 was considered statistically significant.

**Table 2:** Mean of ZEN and α-ZEL concentrations (ng/g) in liver, muscle, and kidney of wild boars from Avellino Province (Campania region of Southern Italy, 2021–2022).

Mycotoxin	Sample	Positive	%	SE%	95% CI	Chi-Square	*p*	Mean Concentration (ng/g)	SE	Kruskal–Wallis Statistic	*p*
	Liver	34	41.5	10.6	30.8–52.1	15.056		1.71	0.339		
ZEN	Muscle	21	25.6	9.5	16.2–35.2		0.0005	1.49	0.493	16.46 *	0.0003 ***
	Kidney	12	14.6	7.7	6.9–22-3			0.65	0.26		
	Liver	16	19.5	8.5	10.9–20.1			0.65	0.2409		
α-ZEL	Muscle	11	13.4	7.4	6.0–20.8	1.972	0.3730	0.66	0.1706	1.218	0.5439
	Kidney	10	12.2	7.1	5.1–19.3			0.77	0.311		

SE: standard error; asterisk: indicates the level of significance; * *p* < 0.05 was considered statistically significant; *** *p* < 0.001.

**Table 3:** Spearman correlation coefficient analysis shows the correlation between the concentrations of ZEN and α-ZEL in liver, muscle, and kidney in relation to the body weight and age of the sampled wild boars.

Variable	Mycotoxin	Organ	Mean Concentration (ng/g)	SD	r	95% CI	*p*
Body Weight							
		Liver	1.716	3.065	0.004	−0.213–0.221	0.9727
	ZEN	Muscle	1.495	4.467	−0.023	−0.239–0.195	0.8338
		Kidney	0.652	2.352	0.020	−0.198–0.236	0.8604
		Liver	0.657	2.174	−0.078	−0.029–0.141	0.4813
	α-ZEL	Muscle	0.666	1.540	−0.072	-0.284–0.148	0.5193
		Kidney	0.770	2.807	−0.077	−0.289–0.143	0.4908
Age							
		Liver	1.716	3.065	0.006	−0.211–0.223	0.9539
	ZEN	Muscle	1.495	4.467	−0.104	−0.314–0.115	0.3477
		Kidney	0.652	2.352	−0.073	−0.286–0.146	0.5099
		Liver	0.657	2.174	−0.017	−0.233–0.201	0.8804
	α-ZEL	Muscle	0.666	1.540	−0.059	−0.273–0.160	0.5951
		Kidney	0.770	2.807	−0.066	−0.279–0.153	0.5505

SE: standard error; *p* < 0.05 was considered statistically significant.

## Data Availability

The data sets used and/or analyzed during the current study are available from the corresponding author.

## Supplementary Materials

The following supporting information can be downloaded at: https://www.mdpi.com/article/10.3390/toxins15010056/s1, Table S1: Validation parameters of HPLC method; LOD = limit of detection, LOQ = limit of quantification, r2 = coefficient of correlation, SD = standard deviation, RSD = relative standard deviation.
