# Supplementary material for: Zearalenone (ZEN) and Its Metabolite Levels in Tissues of Wild Boar (Sus scrofa) from Southern Italy: A Pilot Study
Source: Toxins (Basel). 2023 Jan 9;15(1):56. doi: 10.3390/toxins15010056 (PMC9864771; doi:10.3390/toxins15010056)
Supplement: Supplementary file 1 [file toxins-15-00056-s001.zip › toxins-2111156-supplementary.pdf]

Supplementary file

Table S1. Validation parameters of HPLC method; (LOD = limit of detection, LOQ = limit of quantification, r2 = coefficient of correlation, SD = standard deviation, RSD = relative standard deviation).

**Parameters**		**Muscle**	**Liver**	**Kidney**
			**ZEL**	
**LOD (μg/kg)**		0.05	0.05	0.05
**LOQ (μg/kg)**		0.10	0.10	0.10
**r^2**		0.998	0.996	0.995
**Repeatability**				
0.1	Mean concentration ± SD	0.064 ± 0.004	0.068 ± 0.003	0.069 ± 0.001
	RSD (%)	6.30	3.89	1.67
1.0	Mean concentration ± SD	0.76 ±0.01	0.70 ± 0.02	0.72 ± 0.03
	RSD (%)	2.00	2.47	4.03
5.0	Mean concentration ± SD	3.58 ± 0.02	3.60± 0.05	3.62 ± 0.07
	RSD (%)	0.65	1.25	2.00
**Reproducibility**				
0.1	Mean concentration ± SD	0.064 ± 0.003	0.067 ± 0.002	0.069 ± 0.001
	RSD (%)	4.56	3.33	1.74
1.0	Mean concentration ± SD	0.75 ± 0.02	0.73 ± 0.04	0.73± 0.02
	RSD (%)	2.90	5.14	3.46
5.0	Mean concentration ± SD	3.65 ± 0.08	3.62± 0.03	3.60 ± 0.05
	RSD (%)	2.30	1.63	1.50
**Recovery %**				
0.1		63.67 ± 2.87	66.83 ± 2.30	69.33 ± 1.21
1.0		74.67 ± 2.16	73.17 ± 3.76	73.00 ± 2.53
5.0		71.33 ± 1.62	72.43 ± 0.89	71.93 ± 2.09
			**α-ZEL**	
**LOD (μg/kg)**		0.05	0.05	0.05
**LOQ (μg/kg)**		0.10	0.10	0.10
**r^2**		0.992	0.993	0.995
**Repeatability**				
0.1	Mean concentration ± SD	0.075 ± 0.004	0.067 ± 0.002	0.069 ± 0.002
	RSD (%)	5.58	3.12	3.83
1.0	Mean concentration ± SD	0.73 ±0.02	0.69 ± 0.01	0.70 ± 0.01
	RSD (%)	2.86	2.20	1.50
5.0	Mean concentration ± SD	3.71 ± 0.03	3.63± 0.05	3.75 ± 0.05
	RSD (%)	1.86	1.59	2.60
**Reproducibility**				
0.1	Mean concentration ± SD	0.072 ± 0.004	0.068 ± 0.002	0.070 ± 0.003
	RSD (%)	5.07	3.77	4.88
1.0	Mean concentration ± SD	0.72 ± 0.03	0.71 ± 0.05	0.73± 0.05
	RSD (%)	2.89	3.86	4.12
5.0	Mean concentration ± SD	3.68 ± 0.05	3.59± 0.06	3.71 ± 0.08
	RSD (%)	2.46	2.72	3.14
**Recovery %**				
0.1		72.50 ± 3.68	67.50 ± 2.87	70.33 ± 2.73
1.0		71.67 ± 2.96	71.33 ± 2.73	69.83 ± 1.56
5.0		73.47 ± 2.30	71.80 ± 1.24	74.27 ± 2.58
			**β-ZEL**	
**LOD (μg/kg)**		0.10	0.10	0.10
**LOQ (μg/kg)**		0.25	0.25	0.25
**r^2**		0.993	0.996	0.991
**Repeatability**				
0.1	Mean concentration ± SD	0.063 ± 0.002	0.074 ± 0.003	0.070 ± 0.002
	RSD (%)	4.02	4.45	2.85
1.0	Mean concentration ± SD	0.75 ±0.02	0.73 ± 0.03	0.68 ± 0.03

	RSD (%)	2.86	2.20	1.50
5.0	Mean concentration ± SD	3.61 ± 0.05	3.64± 0.05	3.59 ± 0.08
	RSD (%)	2.39	2.88	3.63
**Reproducibility**				
0.1	Mean concentration ± SD	0.063 ± 0.002	0.072 ± 0.005	0.071 ± 0.003
	RSD (%)	3.01	6.01	4.37
1.0	Mean concentration ± SD	0.74 ± 0.04	0.74 ± 0.03	0.69± 0.05
	RSD (%)	4.58	3.36	4.59
5.0	Mean concentration ± SD	3.64 ± 0.06	3.66± 0.04	3.60 ± 0.05
	RSD (%)	2.65	2.06	2.45
**Recovery %**				
0.1		63.00 ± 3.89	72.43 ± 4.35	72.50 ± 3.20
1.0		74.50 ± 2.67	73.83 ± 2.48	69.17± 2.48
5.0		72.70 ± 2.20	73.90 ± 1.77	73.13 ± 3.33
